# Integrating palliative care education in pulmonary rehabilitation: a randomized controlled study protocol

**DOI:** 10.1186/s12904-024-01363-0

**Published:** 2024-03-20

**Authors:** M. Aurora Mendes, Daisy J. A. Janssen, Alda Marques

**Affiliations:** 1 Pneumologia, Centro Hospitalar do Baixo Vouga (CHBV), Aveiro, Portugal; 2 Respiratory Research and Rehabilitation Laboratory (Lab3R), School of Health Sciences (ESSUA), University of Aveiro, Aveiro, Portugal; 3 Institute of Biomedicine (iBiMED), University of Aveiro, Aveiro, Portugal; 4 Department of Health Services Research, Faculty of Health Medicine and Life Sciences, Maastricht University, Maastricht, The Netherlands; 5 Department of Family Medicine, Faculty of Health Medicine and Life Sciences, Care and Public Health Research Institute, Maastricht University, Maastricht, The Netherlands; 6 Department of Research & Development, Ciro, Horn The Netherlands

**Keywords:** Chronic obstructive pulmonary disease, Interstitial lung diseases, Pulmonary rehabilitation, Palliative care, Education, Family and/or friend caregivers

## Abstract

**Background:**

Palliative care addresses multiple unmet needs of people with chronic obstructive pulmonary disease (COPD) or interstitial lung diseases (ILD) and their family and/or friend caregivers, but it remains highly underused. Pulmonary rehabilitation (PR) may provide a key opportunity to introduce palliative care. We aim to explore the effects of palliative care education as part of PR on knowledge about this field in people with COPD or ILD and their family and/or friend caregivers.

**Methods:**

A randomized controlled study will compare PR with palliative care education (experimental) with traditional PR (control) in people with COPD or ILD and their family and/or friend caregivers. Family and/or friend caregivers will be invited to take part in education and psychosocial support sessions. In addition to the usual educational content, the experimental group will have a session on palliative care, a “Peer-to-peer session”, two “Get-apart sessions” and online sessions. The “Peer-to-peer session” and the “Get-apart sessions” will be discussions about topics suggested by participants. The “Get-apart sessions” will be dedicated to people with COPD or ILD apart from their family and/or friend caregivers and vice versa. The online sessions will be zoom meetings to discuss any health-related issues raised by participants, at a flexible time. A mixed-methods approach will be used to evaluate the outcomes. The primary outcome will be knowledge about palliative care. Secondary outcomes will include attitude towards palliative care referral, symptoms, disease impact, health-related quality of life, needs, knowledge about the disease, burden of providing care, adherence, adverse events and referral to a specialist palliative care team. Quantitative and qualitative data will be collected at baseline and end of PR. At 6-months post-PR, only patient-reported outcomes will be collected. For the primary outcome, time*group interaction will be analyzed with mixed analysis of variance.

**Discussion:**

This study aims to demonstrate the impact of integrating palliative care into the PR education program.

**Trial registration:**

The trial was registered in the ClinicalTrials.gov U.S. National Library of Medicine, on 1st September, 2023 (NCT06046547).

## Background

Chronic respiratory diseases, including chronic obstructive pulmonary disease (COPD) and interstitial lung diseases (ILD) are among the most common non-communicable diseases worldwide [1]. They are leading causes of mortality and constitute 4.1% of disability-adjusted life-years (DALYs) for all causes, with a significant and increasing individual, societal and economic burden [1, 2].

People with COPD or ILD suffer from physical and psychological symptom burden, multiple limitations in activities and restrictions in participation in daily life, and uncertain expectations about the future, which all have a tremendous impact on their health-related quality of life (HRQoL) [3–8]. Family and/or friend caregivers are their main source of support, throughout the whole disease trajectory [5]. They fulfill many roles such as housekeeping and shopping, encouraging adherence to treatments, assisting to coordinate care across providers and settings, recognizing and managing emergency situations and providing emotional and spiritual support over time [5, 9, 10]. Indeed, they facilitate the overcoming of daily challenges and contribute to improved health outcomes e.g., reduce hospitalizations [10, 11]. So, not surprisingly, caring for a person with COPD or ILD can be highly demanding and has major influence on various domains of family and/or friend caregivers’ lives, including physical, psychological, social and financial [5, 12]. In daily clinical practice, this impact of the caring role is undervalued and not addressed by healthcare professionals. In fact, there is limited literature on interventions that include family and/or friend caregivers of people with COPD or ILD [9, 13–17].

Pulmonary rehabilitation (PR) and palliative care are two multicomponent interventions crucial for people with COPD or ILD irrespective of their prognosis [3, 7, 18, 19]. They have been recognized as “two sides of the same coin” [20], by sharing several key characteristics such as: 1) specific aims of managing symptoms, optimizing functional independence and improving HRQoL; 2) a holistic and person-centered approach; 3) individualized goal setting; 4) family and/or friend caregivers involvement and support; and 5) access based on needs [3, 18]. Differences have also been described, namely in the type of treatment interventions used. For example, exercise training is a core component of PR and infrequently used in palliative care. On the other hand, advance care planning is an important element in palliative care and scarcely discussed in PR, even nowadays [3].

Recognizing family and/or friend caregivers as a cornerstone of the unit of care has been consistently advocated by palliative care in different chronic progressive diseases [7, 21] and is clearly stated in the most recent recommendations for serious respiratory illness [18, 22]. Less explored has been the role of PR to support family and/or friend caregivers [9, 13, 15–17]. To date, only one randomized controlled trial has been performed in this area [17]. This study showed that involving the family in PR improves the coping strategies and the psychosocial adjustment to illness of the whole family (not only people with COPD).

In recent years, universal PR and upstream palliative care have been advocated, based on unmet needs [3, 18, 22]. Needs of patients and family and/or friend caregivers living with COPD or ILD encompass multiple life dimensions and include e.g., validation and support throughout the disease, public awareness, need to act and be treated normally, meeting others in similar circumstances, coping strategies, open communication, be included in decision-making, understand the disease, strategies for social participation, support from others but allowing autonomy and medication management/equipment handling [5, 6, 23, 24]. However, palliative care remains highly underused in people with chronic respiratory diseases [18]. One of the reasons reported is the unclear awareness of the potential role of palliative care allied to inadequate knowledge and misperceptions of this field among patients [18, 25, 26].

PR could be an ideal setting to identify and assess unmet needs of people with COPD or ILD and their family and/or friend caregivers and introduce palliative care [3, 19, 27]. Education and psychosocial support sessions are a well-established component of PR. Including palliative care educational content (e.g., concept, role and goals of palliative care, symptom control, disease impact, psychosocial support and planning for the future) in these sessions and actively involving family and/or friend caregivers represents an important opportunity to introduce palliative care in PR. Indeed, already a decade ago advance care planning was mentioned as one of the educational topics in PR [28], which has been well received by patients [29]. Moreover, across the PR program, individuals could be timely referred to a specialist palliative care team, which has the potential to add particular benefit in breathlessness management, advance care planning and psychosocial support. Another possible strategy to improve patients’ and family and/or friend caregivers’ care is educating and training rehabilitation staff in palliative care issues and advanced communication skills [3, 7, 22, 27]. In fact, placing focus on palliative care in PR has the potential to provide the best chance of timely addressing unmet needs of those with COPD or ILD and their family and/or friend caregivers. However, the direct effects of this integrated care model on patients’ and family and/or friend caregivers’ outcomes have not been studied before.

We hypothesize that by integrating palliative care education in PR for people with COPD or ILD and their family and/or friend caregivers we will improve knowledge about palliative care. Furthermore, we hypothesize that by enhancing educational opportunities, namely including the topic of palliative care in PR we will change the attitude towards palliative care referral, improve symptoms, disease impact, HRQoL, needs, knowledge about the disease and reduce burden of providing care.

The primary aim of this study is to explore the effects of palliative care education as part of PR on people with COPD or ILD and their family and/or friend caregivers’ knowledge about palliative care. Secondary aims are to: a) understand the perspectives of people with COPD or ILD and their family and/or friend caregivers about integrating palliative care education in PR; and b) explore the effects of PR with more educational opportunities, namely including the topic of palliative care on people with COPD or ILD and their family and/or friend caregivers’ attitude towards palliative care referral, symptoms, disease impact, HRQoL, needs, knowledge about the disease and burden of providing care.

This article describes the study protocol in detail and provides an overview of its strengths and limitations.

## Methods

### Study design

A randomized controlled study to compare PR with palliative care education (experimental) with traditional PR (control) in people with COPD or ILD and their family and/or friend caregivers has been deigned. The study was registered in the ClinicalTrials.gov U.S. National Library of Medicine (NCT06046547) and will follow the Consolidated Standards of Reporting Trials (CONSORT) statement for nonpharmacological treatments [30], Template for Intervention Description and Replication (TIDieR) checklist and guide [31], Consolidated Criteria for Reporting Qualitative Research (COREQ) guidelines [32] and Good Reporting of A Mixed Methods Study (GRAMMS) framework [33].

Participants will be randomized into an experimental group (EG) and a control group (CG), using a 1:1 allocation ratio and random block sizes of two and four. An independent researcher will use an online software tool (https://www.sealedenvelope.com/simple-randomiser/v1/lists) to generate a computer-based random allocation list and will inform the lead researcher (MAM) about their group allocation via phone call.

Assessors and the researcher responsible for moderating the focus groups will not be involved in PR provision and will be blinded to group allocation. Participants and the researcher conducting the intervention will not be blinded to group allocation, due to the nature of the intervention. The blindness of the researcher performing the data analysis will be ensured by anonymizing all data.

### Participants

People with COPD or ILD and their family and/or friend caregivers will be recruited from the outpatient pulmonary rehabilitation unit at Centro Hospitalar do Baixo Vouga (CHBV), a secondary hospital. Recruitment started in August 2023, with final data collection expected to be completed in June 2026. The pulmonologist of the PR program will identify eligible participants and explain the study. Patients will be eligible if they are adults with a diagnosis of COPD according to the Global Initiative for Chronic Obstructive Lung Disease (GOLD) criteria [34] or with a multidisciplinary diagnosis of ILD [35] and are clinically stable in the previous month (i.e., without acute exacerbation). Patients will be excluded if they present a musculoskeletal, neurological or psychiatric condition which may limit their participation in PR, had participated in any PR program in the last 6 months, had received specialist palliative care in the last 12 months or are unable to understand Portuguese. Family and/or friend caregivers will be eligible if they are adults identified by the participating people with COPD or ILD as caregivers. For this purpose, it will be explained to people with COPD or ILD that a family and/or friend caregiver is any relative, partner, friend, neighbor, or significant other with personal relationship with them, and who provides a broad range of unpaid assistance, namely with activities of daily living (e.g., toileting, feeding and bathing) and instrumental activities of daily living (e.g., shopping, meal preparation and managing finances)] [36]. Family and/or friend caregivers will be excluded if they present a neurological or psychiatric condition which may limit their participation or are unable to understand Portuguese. An appointment with those willing to participate will be scheduled to clarify any doubts, gather informed consents and conduct baseline assessments.

### Intervention

Both groups will follow a multidisciplinary team-based PR model at CHBV delivered by a pulmonologist, a physiotherapist and a nurse, and will include supervised exercise sessions and education and psychosocial support sessions in a group setting over a 12-week period. Other healthcare professionals, including a psychologist and a nutritionist will be involved in coordinating education and psychosocial support sessions.

#### Exercise sessions

The exercise sessions will follow the same structure for both groups and will be delivered in accordance with international guidelines [35, 37, 38]. It will occur twice per week for a maximum of 90 min. Continuous telemetry with electrocardiogram and oxygen saturation will be used and perceived dyspnoea and fatigue will be monitored with modified Borg scale throughout the session [39]. Intensity of the aerobic and resistance training will be prescribed using the 6-min walking test (6-MWT) or the cardiopulmonary exercise testing (if available) and the one-repetition maximum (1-RM), respectively. Exercise sessions will include 10 min of warm up, 30 to 40 min of aerobic cycle ergometer and/or treadmill training at 80% of the average speed achieved during the 6-MWT or 60% of their work peak [37, 40], 20 to 30 min of resistance training at 60 to 70% of 1-RM [37], and 10 min of cool down. Progression of training intensity will be tailored according to the perceived dyspnoea and fatigue (4–6 in the modified Borg scale) [39]. Adjunct components such as airway clearance techniques, balance training, inspiratory muscles training and electric neuromuscular electrical stimulation will be prescribed according to individuals’ needs within this timeframe.

#### Education and psychosocial support sessions

The education and psychosocial support sessions will follow the same structure for both groups, however the number of sessions will be different, and the content areas will sometimes vary, as described below.

Each education and psychosocial support session will be delivered after the exercise training and will last approximately 60 min. Family and/or friend caregivers will be invited and encouraged to take part in all sessions. They will be led by healthcare professionals of the multidisciplinary team and all participants will sit in a circular arrangement. An interactive style will be followed, to engage people with COPD or ILD and their family and/or friend caregivers in an active dialogue and reflection about the topic being discussed. The healthcare professional will avoid medical jargon and facilitate participation of the group e.g., asking open questions that lead to meaningful interaction. An active effort will be conducted to adapt the education process to the characteristics of participants at an individual level e.g., revising topics covered at the end to confirm the achievement of the core knowledge and skills. Moreover, written materials will be provided at the end of each session.

##### Sessions dedicated to both groups

The following topics will be explored in both groups (i.e., EG and CG): a) information on chronic respiratory diseases; b) medication, inhaler techniques, oxygen therapy and non-invasive ventilation; c) symptom management and exacerbations; d) exercise and physical activity; e) action plan; f) anxiety and depression management; and g) nutrition.

##### Sessions dedicated only to the experimental group

For the EG there will be four additional face-to-face sessions: a palliative care session, a “Peer-to-peer session” and two “Get-apart sessions” (i.e., one dedicated exclusively to people with COPD or ILD and another one dedicated exclusively to their family and/or friend caregivers); and online sessions, within the 12-week period.

In “Peer-to-peer session” and “Get-apart sessions” the focus will be to discuss participants’ own issues with the multidisciplinary team but opinions about how to optimize PR for them will also be gathered. Moreover, every two weeks and for approximately 40 min, people with COPD or ILD and their family and/or friend caregivers will have the opportunity to discuss any health-related issues with a healthcare professional from the multidisciplinary team via zoom platform (i.e., online sessions). These sessions will be individualized according to participants learning needs and perspectives identified during PR. Moreover, participants that cannot attend to face-to-face sessions will have this opportunity to discuss the most relevant information to manage their disease.

Individual cases from both groups will also be referred for evaluation by a specialist palliative care team or by any other health and social care professional (e.g., psychologist or social worker) according to the specific unmet needs identified.

##### Education session on palliative care

The education session on palliative care will be facilitated by two healthcare professionals, a medical doctor and a nurse, both specialized in the field. The main topics that will be discussed are presented in Table 1. A written leaflet summarizing the main information about palliative care will be given to people with COPD or ILD and their family and/or friend caregivers.

**Table 1:** Description of the education session on palliative care delivered during pulmonary rehabilitation to people with chronic obstructive pulmonary disease or interstitial lung diseases and their family and/or friend caregivers

Content areas	Discussion topics
Palliative care	Concept/definition of palliative care Role and goals of palliative care
Symptom control	Dyspnoea (including dyspnoea crisis), fatigue, cough and anorexia management
Impact of the disease	Impact of the disease on individual and family and/or friend caregiver Maintaining a balance between hope and fears
Psychosocial support	Hospital- and community-based resources
Planning for the future	Advance care planning

##### “Peer-to-peer session” and “Get-apart sessions”

People with COPD or ILD and their family and/or friend caregivers will be the primary communicators in the “Peer-to-peer session”, and the dialogue will be based on participants suggestions. This session will provide participants with an opportunity to share their knowledge, competences and experiences, listen to other people living with the same disease, and to some extent give and receive support from peers. In the week before, people with COPD or ILD will receive a total of nine cards with printed statements corresponding to the Patient Reported Experience Measure in Chronic Obstructive Pulmonary Disease (PREM-C9) [41] items. People will be asked to select one or two cards of most important personal meaning and value, and the session will be initiated talking about the statements’ themes. This strategy will capture some possible topics that people with COPD or ILD would like to discuss, prioritize content, as well as promote participant engagement.

In the “Get-apart sessions”, people with COPD or ILD will have the opportunity to discuss their issues apart from their family and/or friend caregivers and vice versa. This can be particularly helpful for people who feel more reluctant, ashamed or even stigmatized to reveal private concerns, doubts and thoughts in front of their loved ones. Both the “Peer-to-peer session” and “Get-apart sessions” will be supervised by healthcare professionals from the multidisciplinary team.

#### Referral of individual cases to the specialist palliative care team

Participants from both groups might be referred to the specialist palliative care team by the pulmonologist of the PR program, who has interest and basic training in the field. The decision will be taken after considering the input of all members of the multidisciplinary team and the holistic assessment of the person with COPD or ILD and the family and/or friend caregiver. Multiple aspects will be considered such as symptom severity, indicators of clinical deterioration, functional status decline and respiratory function, as well as family and/or friend caregiver’ needs [18, 22, 42]. People with COPD or ILD and family and/or friend caregivers who manifest willingness to be evaluated by a specialist palliative care team will also be referred. Participants might be referred at any time point during the intervention, and such decision (refer or not refer) will carefully be reviewed at the end of PR.

### Baseline assessments

Quantitative data will be collected from all participants at baseline

The following data will be collected from people with COPD or ILD through self-report, healthcare records and direct measurements: a) sociodemographic information (i.e., age, sex, educational level, marital and employment status); b) health status [smoking status, comorbidity(ies), medication, oxygen treatment, non-invasive ventilation and exacerbation(s), hospital admission(s) and fall(s) within the last 12 months]; c) health literacy (European Health Literacy Survey in Portuguese, HLS-EU-PT) [43]; d) cognitive status (six-item cognitive impairment test, 6CIT) [44]; e) anthropometry [height and weight to calculate the body mass index (BMI)]; and f) respiratory function [residual volume (RV), total lung capacity (TLC), RV/TLC ratio, pre and post bronchodilator forced expiratory volume in the first second (FEV_1_) and forced vital capacity (FVC), and diffusing capacity for carbon monoxide (DLCO)].

Family and/or friend caregivers will provide information on their: a) sociodemographics (i.e., age, sex, educational level, marital and employment status); b) health status [disease(s), limitation(s) in activities of daily living, medication and fall(s) within the last 12 months]; c) health literacy (HLS-EU-PT); d) cognitive status (6CIT); and e) role of caring [relationship with the care receiver, additional help (if any) from other co-caregiver(s), caregiver tasks and amount of time performing these tasks, length of caring, geographical distance to the care receiver house, and single vs multiple care receiver(s) at once].

### Outcomes

The primary outcome will be knowledge about palliative care evaluated with the Palliative Care Knowledge Scale (PaCKS) [45]. Data will be collected at baseline (T0) and end of PR (T1). At 6-months post-PR (T2), only patient-reported outcome measures will be collected. Table 2 provides a detailed description of primary and secondary outcomes that will be assessed throughout the study period. Some outcomes, such as functional capacity, peripheral muscle strength and balance are not expected to be influenced by education, however they are important to assess the effectiveness of PR delivery.

**Table 2:** Overview of outcomes and outcome measures evaluated in people with chronic obstructive pulmonary disease or interstitial lung diseases and their family and/or friend caregivers

Outcome	Outcome measure	Description	MCID^a^	People with COPD or ILD	Family and/or friend caregivers	T0	T1	T2
Knowledge about palliative care	PaCKS [45]	- Self-reported questionnaire with 13 items which are true or false. A broad range of topics are assessed, including goals, target population and timing of palliative care, as well as symptoms and problems that palliative care addresses - For each statement, “True”, “False” and “I don’t know” options are provided, and a mark is given for each correct answer - Scores range from 0/13 to 13/13, with higher scores indicating higher knowledge	Not applicable	⬤	⬤	×	×	×
Attitude towards palliative care referral	“Would you like (your loved one) to be referred to a specialist palliative care team at this time?” [46]	- “Would you like to be referred to a specialist palliative care team at this time?” and “Would you like your loved one to be referred to a specialist palliative care team at this time?” will be asked to people with COPD or ILD and their family and/or friend caregivers, respectively - Possible answers are “Yes”, “No” or “Don’t know”	Not applicable	⬤	⬤	×	×	×
	“Would you like (your loved one) to be referred to a specialist palliative care team if your (your loved one’) health deteriorates?”	- “Would you like to be referred to a specialist palliative care team if your health deteriorates?” and “Would you like your loved one to be referred to a specialist palliative care team if your loved one’ health deteriorates?” will be asked to people with COPD or ILD and their family and/or friend caregivers, respectively who do not answer "Yes" to the previous question - Possible answers are “Yes”, “No” or “Don’t know”	Not applicable	⬤	⬤	×	×	×
Pain	“Do you feel pain?” Pain charts [47] and VAS [48, 49]	- Participants will be asked about pain perception with a closed question: “Do you feel pain?” - In affirmative cases, pain charts and VAS will be used to identify its location and to measure its intensity, respectively - Scores will be recorded on a 10 centimeters (cm) line that represents a continuum between “no pain” and “worst pain”	VAS -1.37 cm	⬤	⬤	×	×	×
Dyspnoea	mMRC [50, 51]	- 5-point scale graded from 0 (“No breathlessness except on strenuous exercise”) to 4 (“Too breathless to leave the house or breathless when dressing or undressing”). Higher scores indicate greater dyspnoea severity on daily activities	-1 grade	⬤		×	×	×
Fatigue	FACIT-FS [52, 53]	- 13-item questionnaire assessing tiredness, weakness, listlessness, lack of energy, and their impact on HRQoL - Each item is rated from 0 (“Not at all”) to 4 (“Very much”). Total score ranges from 0 to 52. Higher scores indicate less fatigue	+ 4.7 points	⬤	⬤	×	×	×
Cough	LCQ [54, 55]	- 19-item questionnaire containing three domains: physical, psychological and social - Each item is rated from 1 to 7. Final score ranges from 3 to 21. Higher scores indicate weaker influence of cough on quality of life	+ 1.3 points	⬤		×	×	×
Anxiety and depression	HADS [56, 57]	- 14 multiple-choice items divided into 7 item subscales for anxiety (HADS-A) and depression (HADS-D) - Scores in each subscale range from 0 to 21. Higher scores indicate greater levels of anxiety and/or depression. Clinically significant anxiety or depression are interpreted by scores ≥ 8	HADS-A -1.5 points HADS-D -1.5 points	⬤	⬤	×	×	×
Disease impact	CAT [58, 59]	- 8-item questionnaire which addresses cough, sputum, chest tightness, dyspnoea, home daily activities, confidence leaving home, sleep and energy levels in a 6-point scale rated from 0 to 5 - Score ranges from 0 to 40. Higher scores indicate more severe impairment on health status. A score of more than 20 indicates high impact	-2 points	⬤		×	×	×
HRQoL	SGRQ [60, 61]	- 50-item questionnaire which evaluates three different domains contributing to overall health, daily life, and perceived well-being: symptoms, activity and impact - A score in each domain and a total score are calculated and weighted, ranging from 0 to 100. Higher scores indicate worse HRQoL	-4 points	⬤		×	×	×
Needs	SNAP [62, 63]	- 15 questions with the format: “Do you need more support with…” (e.g., “Do you need more support with managing your symptoms?”) - For each statement, “No”, “A little more” and “Quite a bit more” options are provided to identify the domains in need of support - There is an optional additional open-ended question to capture “anything else” not already covered - The final question refers to the caregiver needs: “Does a family member or friend who helps you need more support?”	Not applicable	⬤		×	×	×
	CSNAT [64, 65]	- 14 questions covering caregivers’ broad support domains which fall into two distinct groups: support that enable them to provide care and more direct personal support for themselves - All questions follow the format: “Do you need more support with…” (e.g., “Do you need more support with knowing what to expect in the future?”) - For each question, “No”, “A little more”, “Quite a bit more” and “Very much more” options are provided to identify support needed within any of the domains - There is an optional additional open-ended question to capture “anything else” not already covered	Not applicable		⬤	×	×	×
Functional performance	LCADL [66, 67]	- 15 activities of daily living organized in four domains: self-care, domestic, physical and leisure - Each item is rated from 0 (“I wouldn’t do it anyway”) to 5 (“I need someone else to do this”), except for one additional question on global impact with “A lot”, “A little” and “Not at all” as answer choices - Final score ranges from 0 to 75. Higher scores indicate greater functional limitation	LCADL -3 points LCADL % of total -4 points	⬤		×	×	×
Functional capacity	6-MWT [68, 69]	- Evaluates the distance walked at a fast pace during 6 min in a 30 meters (m) corridor	+ 30 m	⬤		×	×	
Peripheral muscle strength	Quadriceps strength 1-RM [37, 70] Handgrip strength HHD [71, 72]	- Quadriceps strength, 1-RM: determines the greatest amount of weight in kilogram (kg) that the participant could move in a double leg extension manoeuvre (isotonic strength) - Handgrip strength, HHD: measures the maximum isometric strength of the hand and forearm muscles at the dominant side in kg	Quadriceps + 5.7 kg, 26.9% Handgrip Not available	⬤		×	×	
Balance	Brief-BESTest [73, 74]	- 8-item scale evaluating 6 domains contributing to postural control in standing and walking: biomechanical constraints, stability limits/verticality, transitions/anticipatory postural adjustments, reactive postural control, sensory orientation and stability gait - Each domain is evaluated with a test scored from 0 (severe impairment) to 3 (no impairment), with a maximum of 24. Higher scores indicate better balance performance	+ 3 points	⬤		×	×	
Knowledge about COPD	BCKD [18]	- 65 statements divided in 13 topics, each with a stem; these topics cover epidemiology and physiology, aetiology, common symptoms, breathlessness, phlegm, chest infections, exercise, smoking, immunization, inhaled bronchodilators, antibiotics, and oral/inhaled steroids - For each statement, “True”, “False” and “I don’t know” options are provided, and a mark is given for each correct answer - Total score corresponds to the percentage of correct answers	Not available	⬤	⬤	×	×	×
Burden of providing care	ZBI [75]	- 22-item questionnaire addressing impact of caring experience in several domains: health and wellbeing, personal and social life, and finances - Each question has five response options rated from 0 (“Never”) to 4 (“Almost always”), except for the final question on global burden, rated from 0 (“Not at all”) to 4 (“Extremely”). Final score ranges from 0 to 88. Higher scores indicate greater burden. A score of more than 24 indicates high burden	Not available		⬤	×	×	×
Adherence	-	- Number of attended exercise sessions and education and psychosocial support sessions	Not applicable	⬤	⬤		×	
Adverse events	-	- Occurrence of adverse events during PR	Not applicable	⬤			×	
Referral to a specialist palliative care team	-	- Number of people with COPD or ILD referred to a specialist palliative care team	Not applicable	⬤			×	×

The symbol ⬤ identifies the outcomes to be assessed in people with chronic obstructive pulmonary disease or interstitial lung diseases and/or their caregivers. The symbol × identifies the outcomes to be assessed in each timepoint (i.e., T0, T1 and T2)

*Legend—T0* Baseline, *T1* End of pulmonary rehabilitation, *T2* 6-months post-pulmonary rehabilitation, *1-RM* One-repetition maximum, *6-MWT* 6-min walking test, *BCKD* Bristol COPD Knowledge Questionnaire, *Brief-BESTest* Brief-Balance Evaluation Systems Test, *CAT* COPD Assessment Test, *COPD* Chronic obstructive pulmonary disease, *CSNAT* Carer Support Needs Assessment Tool, *FACIT-FS* Fatigue Functional Assessment of Chronic Illness Therapy-Fatigue Subscale, *HADS* Hospital Anxiety and Depression Scale, *HHD* Hand-held dynamometer, *HRQoL* Health-related quality of life, *ILD* Interstitial lung diseases, *LCADL* London Chest Activities of Daily Living, *LCQ* Leicester Cough Questionnaire, *MCID* Minimal clinically important difference, *mMRC* Modified Medical Research Council questionnaire, *PaCKS* Palliative Care Knowledge Scale, *PR* Pulmonary rehabilitation, *SGRQ* Saint George’s Respiratory Questionnaire, *SNAP* Support Needs Approach for Patients, *VAS* Visual analogue scale, *ZBI* Zarit Burden Interview

^a^The minimal clinically important differences (MCID) reported are based on data from people with chronic obstructive pulmonary disease, except the MCID of visual analogue scale (VAS) that is based on data from people with rheumatological diseases

### Focus groups

Qualitative data will be collected only from the EG, at baseline and end of PR. Each focus group will last approximately 90 min and will be audio-recorded and transcribed verbatim, with field notes taken to capture participants’ nonverbal expressions. Participant’s identifying information will not be recorded on the transcripts. We will attribute fictitious names to each participant to ensure anonymity. Interview guides using open-ended questions were developed to explore: a) pre-intervention – knowledge, perceptions, experiences and perceived needs of palliative care (e.g., “What words do you associate with palliative care?”; “Can you remember the first time that you heard about palliative care?”, in affirmative cases: “Please, share your experience. When was it? What did it mean to you?”); and general opinion on integrating palliative care education in PR; and b) post-intervention – acceptability, perceived benefits, added value and impact of integrating palliative care education in PR (e.g., “When do you think is the most appropriate time for assessment by a specialist palliative care team?” and “Did the integration of palliative care education in the PR program had any impact on your physical, psychosocial and spiritual well-being?”); and suggestions for its improvement. The researcher moderating the focus groups will be responsible to get all participants to talk and fully explain their answers, remaining neutral and listening attentively with sensitivity and empathy.

### Sample size calculation

The sample size was estimated for the primary outcome measure, PaCKS, in G*Power 3.1.9.4, for the time*group interaction of a mixed analysis of variance (ANOVA) with two groups (control and experimental) and two timepoints (at baseline and end of PR). We considered an α of 0.05, a power of 0.80, a correlation among repeated measures of 0.5, a nonsphericity correction of 1 and an expected effect size f of 0.25 [76]. The calculated sample size was 34 and considering a possible 40% dropout and missing data rate [77], the final sample size was determined to be 58 (29 in each group).

### Statistical analysis plan

A mixed-methods approach [78] will be used to explore the effects of the intervention. First, quantitative and qualitative data will be analyzed separately, in a parallel process.

Quantitative data will be processed using IBM SPSS Statistics version 29.0 and RStudio 2022.12.0. Categorical variables will be presented as counts(percentages). Quantitative variables will be summarized using mean ± standard deviation or median[interquartile range], based on the normality of data distribution which will be explored with Shapiro–Wilk test. The EG will be compared with the CG for the primary outcome with a mixed ANOVA if assumptions are met, followed by a multiple pairwise comparison with Bonferroni correction; otherwise, an aligned rank transform-ANOVA will be performed instead. Imputation methods will be used to deal with missing data as appropriate, followed by sensitivity analyses. For all statistical analyses, a *p* value < 0.05 will be considered statistically significant.

Qualitative data will be processed using Web Qualitative Data Analysis (WebQDA) for inductive thematic analysis [79]. The proposed six-step procedure will be followed: 1. familiarizing with the data; 2. generating initial codes; 3. searching for themes; 4. reviewing themes; 5. defining and naming themes; and 6. producing the report [79]. Trustworthiness of the qualitative research will be ensured using the credibility, transferability, dependability, and confirmability criteria [80]. Credibility will be ensured by a) researcher triangulation i.e., each focus group will be transcribed and preliminary coded by one researcher (MAM); then, data will be independently analyzed by two researchers (MAM and AM), which will agree on themes and subthemes; and finally, all the research team (MAM, AM and DJ) will review the themes and subthemes until consensus is reached; and, b) engagement with data by all the research team. Transferability will be ensured by a comprehensive and detailed description of the research setting and participants. Dependability will be ensured by having an independent researcher that will examine, explore and challenge the processes of data collection, analysis and interpretation. Confirmability will be ensured by researcher reflexivity, peer debriefing and member checking among people with COPD or ILD and their family and/or friend caregivers.

Afterwards, we will compare quantitative and qualitative data and merge them through joint displays for integration of both methods in the final interpretation of the results.

## Discussion

Living with COPD or ILD imposes enormous daily challenges, especially at advanced stages, not just to patients but also to family and/or friend caregivers. Their needs are not fully addressed by disease-modifying treatments. A key strategy to improve their well-being is the early integration of palliative care into routine management of COPD and ILD [14, 18, 22]. The current study is designed to explore the effects of palliative care education as part of PR in people with COPD or ILD and their family and/or friend caregivers.

### Strengths

This study has several strengths that should be acknowledged. First, it is an innovative approach in PR dedicated to the person with COPD or ILD/caregiver dyad. Second, the education session on palliative care will be led by a medical doctor and a nurse with expertise in this field and directly involved in the session design. Third, a variety of teaching methods will be used (e.g., interactive lecturing and written material) which may enhance the capacity of learning. Fourth, the availability of an education session directed to each stakeholder is an opportunity to personalize care at an individual level and to tailor support to their distinct needs and experiences [5]. Fifth, online sessions with a flexible meeting schedule may minimize temporal and geographical barriers (e.g., work schedule and/or lack of transportation) to engage in face-to-face education and psychosocial support sessions. Sixth, the study will be described according to the most relevant guidelines [30–33], to allow its replication. Finally, a mixed-methods approach with complementary strengths of the quantitative and qualitative data will provide in-depth understanding of the effects of this intervention [78].

### Limitations

This study has some limitations that should be considered. First, it is a single centre study, hence, results may not be generalized to other contexts (i.e., potentially limited external validity). Second, the heterogeneity of people with COPD or ILD can impact the main findings of the study. Nevertheless, some similarities across experiences and needs of people with different chronic respiratory diseases and their family and/or friend caregivers seem to exist. Therefore, it is likely that they will all benefit from the intervention. Third, recruiting family and/or friend caregivers, that are rarely included in PR, may be difficult. Family and/or friend caregivers are frequently active people with a multiplicity of roles often also unaware of the potential benefits of PR for the person with COPD or ILD and for the whole family. These circumstances may result in reluctance to participate in the study. However, we will use some strategies to minimize this limitation, such as explore potential barriers to participation, be flexible with online sessions and appeal to their fundamental role in disease management intervention studies. Finally, participants and the researcher conducting the intervention will not be blinded, which may influence the results.

### Clinical implications

This study may be a first step towards a greater personalization of PR which integrates palliative care education to people with COPD or ILD and their family and/or friend caregivers. If successful, it will provide preliminary evidence of an innovative model that involves family and/or friend caregivers as pivotal players in daily clinical management of COPD and ILD and includes palliative care education as a core component of PR.

## Data Availability

Not applicable.

## Abbreviations

1-RM: One-repetition maximum
6CIT: Six-item cognitive impairment test
6-MWT: 6-minute walking test
BCKD: Bristol COPD Knowledge Questionnaire
BMI: Body mass index
Brief-BESTest: Brief-Balance Evaluation Systems Test
CAT: COPD Assessment Test
CG: Control group
COPD: Chronic obstructive pulmonary disease
CSNAT: Carer Support Needs Assessment Tool
DAILYs: Disability-adjusted life-years
DLCO: Diffusing capacity for carbon monoxide
EG: Experimental group
FACIT-FS: Fatigue Functional Assessment of Chronic Illness Therapy-Fatigue Subscale
FEV_1_: Forced expiratory volume in the first second
FVC: Forced vital capacity
HADS: Hospital Anxiety and Depression Scale
HHD: Hand-held dynamometer
HRQoL: Health-related quality of life
ILD: Interstitial lung diseases
LCADL: London Chest Activities of Daily Living
LCQ: Leicester Cough Questionnaire
MCID: Minimal clinically important difference
mMRC: Modified Medical Research Council questionnaire
PaCKS: Palliative Care Knowledge Scale
PR: Pulmonary rehabilitation
PREM-C9: Patient Reported Experience Measure in Chronic Obstructive Pulmonary Disease
RV: Residual volume
SGRQ: Saint George’s Respiratory Questionnaire
SNAP: Support Needs Approach for Patients
TLC: Total lung capacity
VAS: Visual analogue scale
ZBI: Zarit Burden Interview
